# CT Ventriculography for diagnosis of occult ventricular cysticerci

**DOI:** 10.4103/2152-7806.74188

**Published:** 2010-12-23

**Authors:** Sebastian R. Herrera, Michael Chan, Ali M. Alaraj, Sergey Neckrysh, Michael G. Lemole, Sepideh Amin-Hanjani, Konstantin V. Slavin, Fady T. Charbel

**Affiliations:** Department of Neurosurgery, University of Illinois at Chicago, Chicago, IL 60612; 1 Department of Surgery, Division of Neurosurgery, University of Arizona, Tucson, AZ 85724

**Keywords:** Intraventricular cyst, hydrocephalus, neurocysticercosis, ventriculography

## Abstract

**Background:**

Neurocysticercosis is the most common parasitic infection of the central nervous system (CNS). Intraventricular lesions are seen in 7–20% of CNS cysticercosis. Intraventricular lesions can be missed by computed tomography (CT) and magnetic resonance imaging (MRI) as they are typically isodense/isointense to the cerebrospinal fluid. We present our experience with CT ventriculography to visualize occult cysts.

**Case Description:**

Two patients presented with hydrocephalus and suspected neurocysticercosis were evaluated with CT and MRI with and without contrast failing to reveal intraventricular lesions. CT-ventriculography was used: 10 ml of cerebrospinal fluid was drained from the ventriculostomy catheter, and 10 ml of iohexol 240 diluted 1:1 with preservative-free saline was injected through the ventriculostomy catheter. Immediate CT of the brain was performed. The first patient had multiple cysts located throughout the body of the left lateral ventricle. The second patient had a single lesion located in the body of the lateral ventricle. The CT-ventriculography findings helped in identifying the lesions and plan the surgical intervention that was performed with the aid of an endoscope to remove the cysts.

**Conclusions:**

Intraventricular neurocysticercosis is a common parasitic disease which can be difficult to diagnose. We used CT-ventriculography with injection of contrast through the ventriculostomy catheter in two patients where CT and MRI failed to demonstrate the lesions. This technique is a safe and useful tool in the imaging armamentarium when intraventricular cystic lesions are suspected.

## INTRODUCTION

Neurocysticercosis is the most common central nervous system (CNS) parasitic infection worldwide.[1–3,6,7,12–14,17] Frequency of CNS involvement may be as high as 60–90% of the cysticercosis cases,[2,3,6] often presenting with seizures, cognitive decline and hydrocephalus (HCP).[2,9,16,17] Intraventricular extension of the disease is seen in 7–20% of the neurocysticercosis cases.[3,6,8,12,13] This location has been associated with worse outcome.[4,8]

Intraventricular lesions are usually diagnosed by computer tomography (CT) or magnetic resonance imaging (MRI). However, cystic intraventricular lesions are often missed by CT because they are isodense to cerebrospinal fluid (CSF) and do not enhance with contrast.[8,16] MRI is the preferred imaging modality, revealing up to 50–80% of the intraventricular lesions.[6,8,11,14] We present our experience with intraventricular injection of contrast material through ventriculostomy catheters followed by immediate CT imaging as an alternative sensitive imaging modality to visualize cysts that are not apparent on routine CT or MR.

## CASE DESCRIPTIONS

### Technique for CT ventriculography

Ventriculostomy catheters (Hermetic-Ventricular catheter set – INS 0001, Integra Life, Plainsboro, NJ) previously implanted for management of acute hydrocephalus were used for ventriculography. Prior to the injection, CT of the head was performed to confirm that all side ports of the catheter were within the lateral ventricle. The patient was positioned supine in the CT scanner in preparation for an immediate scan. Ten milliliter of CSF was passively drained into the collection chamber in the CSF drainage system (Integra, Plainsboro, NJ) prior to the contrast injection. The side port of the drainage system was accessed with a 23-gauge needle while using standard antiseptic technique. Ten milliliter of equal mixture of iohexol 240 (GE Healthcare, Waukesha, WI) and preservative-free saline was injected slowly through the ventriculostomy catheter. Immediate CT of the brain was performed to improve visualization of the entire ventricle as the contrast media settles toward the dependent portion of the ventricle with gravity.

### Case 1

**Figure 1:**
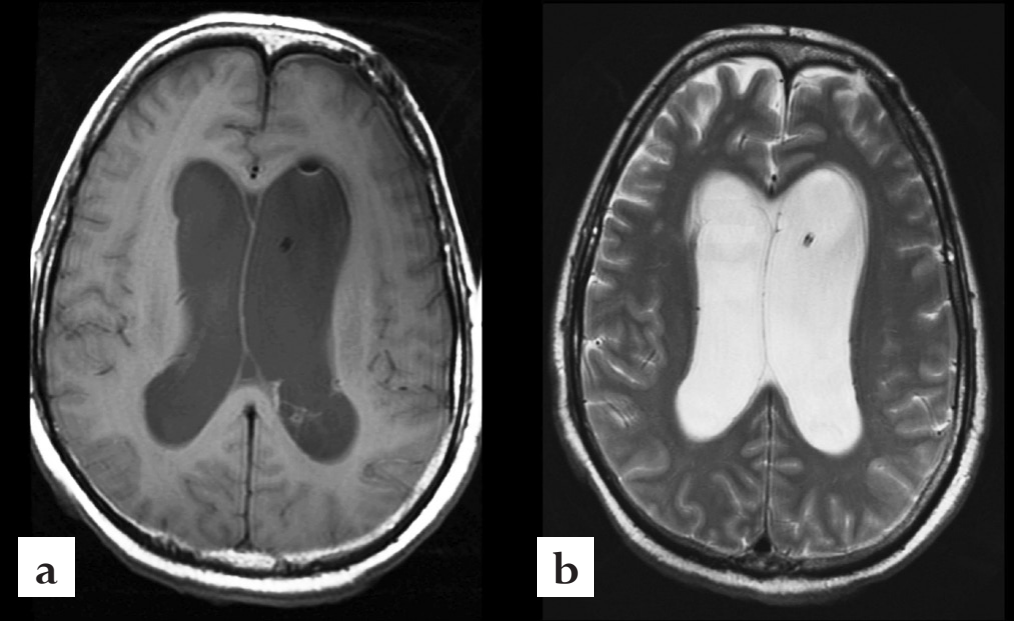
Case #1. (a): Axial T1 weighted magnetic resonance imaging of the brain without contrast showing no intraventricular lesions. (b): Axial T2 weighted magnetic resonance imaging showing ventriculostomy catheter in the body of left lateral ventricle.

**Figure 2:**
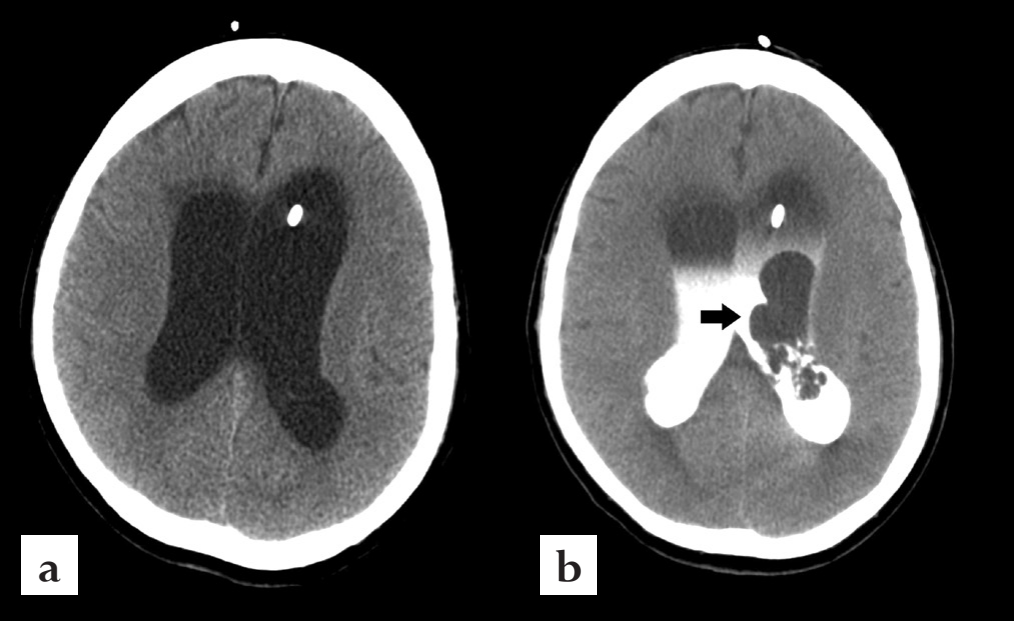
Case #1. (a): CT scan of the brain without contrast showing L frontal ventriculostomy in lateral ventricle. (b): CT scan of the brain during injection of intraventricular iohexol showing multiple lesions in the body of the left lateral ventricle.

A 40-year-old Hispanic male was transferred to our service with a history of neurocysticercosis, hydrocephalus and ventriculoperitoneal shunt malfunction. The patient had previously undergone multiple shunt revisions and arrived on our service with a ventriculostomy catheter in place. CT without contrast failed to demonstrate intraventricular lesions. MRI raised suspicion for a T2 hyperintense lesion within the left lateral ventricle [Figure 1]. CT ventriculography was performed for better visualization of the intraventricular space. This study revealed multiple cystic lesions throughout the body, atrium and occipital horn of the ventricle that was injected [Figure 2]. The imaging also revealed no obstruction at the foramen of Monro as the contralateral and third ventricle filled with contrast. The medical treatment in this case was consistent of oral antihelmintic agent together with supportive measures including multiple shunts for treatment of hydrocephalus. The surgery was recommended and undertaken because of persistence of symptomatic cysts despite completion of standard medical treatment.

### Case 2

**Figure 3:**
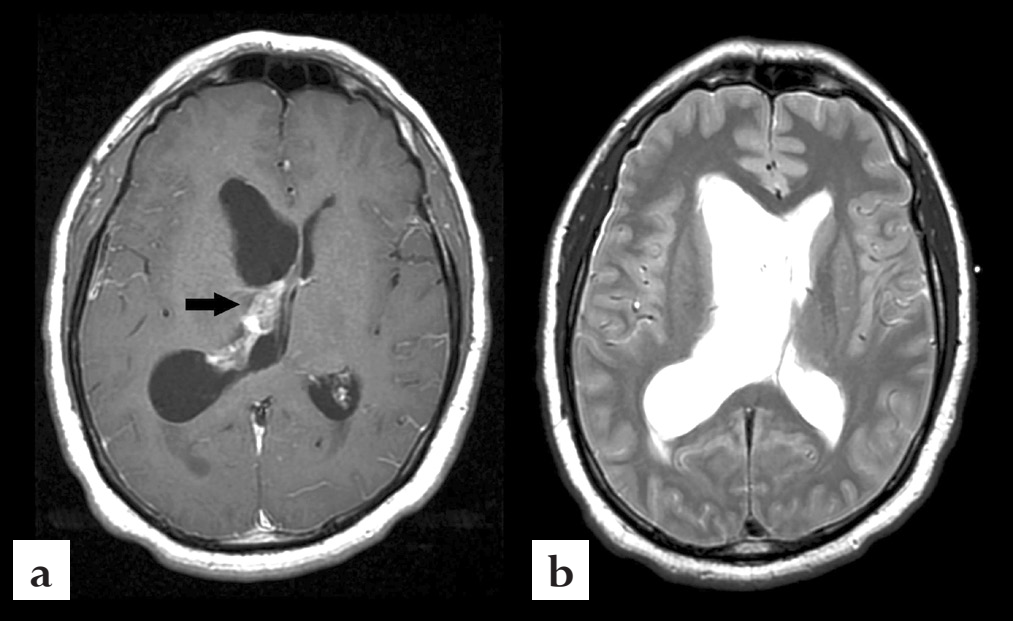
Case #2. (a): Axial T1 with contrast magnetic resonance imaging of the brain showing enhancement in right lateral ventricle without evidence of intraventricular cysts. (b): Axial T2 weighted magnetic resonance imaging of the brain showing right ventriculostomy catheter in the lateral ventricle. No intraventricular cysts were seen.

**Figure 4:**
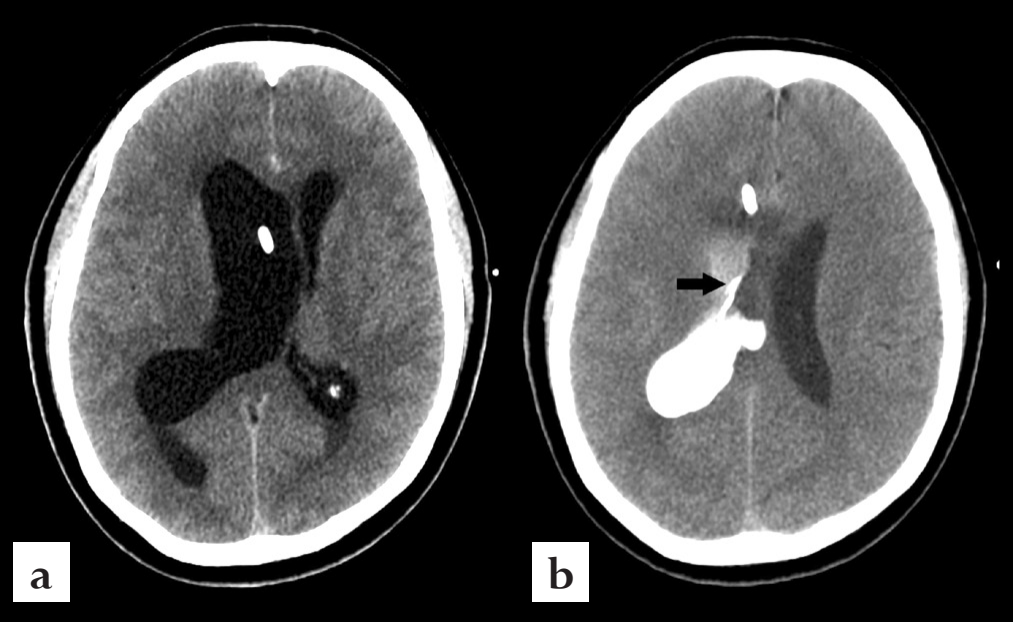
Case # 2. (a): Axial CT scan of the brain without contrast showing right ventriculostomy catheter in the lateral ventricle. (b): Axial CT scan of the brain with ventricular injection of iohexol showing the outline of the right ventricular lesion.

A 41-year-old Hispanic male presented to the emergency department with two weeks of progressive headaches, nausea and vomiting. His neurological exam was non-focal and the initial CT scan showed right lateral ventricle dilatation. A ventriculostomy was placed for management of hydrocephalus. MRI of brain failed to show lesions on T2 and T2 FLAIR weighted sequences, but a suspected cyst was visualized in the T1 with contrast sequence. [Figure 3] In order to confirm the findings, CT-ventriculography was performed revealing the location of a single cyst and obstruction of the foramen of Monro as no contrast reached the third ventricle. [Figure 4] At a later date, the patient was taken for endoscopic surgery using the ventriculostomy tract and the ventricular lesion was removed. Diagnosis was made on CSF sampling. The patient was treated with albendazole and steroids.

## DISCUSSION

Neurocysticercosis is the most common parasitic infection that affects the CNS.[1–3,6,7,13,14,17] This disease is endemic in certain parts of the world and in Latin America, there are approximately 400,000 cases of symptomatic disease.[4,14] Seizures, seen in up to 70% of the cases, are the most common manifestation; however this may vary according to the location of the parasite within the CNS. Locating intraventricular lesions is critical as this form of the disease has been associated with worse outcome.[4,8]

Four stages of the disease have been proposed: vesicular, colloid vesicular, granular nodular and nodular calcified.[14,16] In the vesicular stage, the parasite is alive and is not well seen as the cyst fluid has the same intensity as the CSF. The colloidal stage is seen when the fluid turns thicker and the scolex is degenerating causing perilesional inflammation and contrast enhancement. As the scolex dies, the granular nodular stage takes place. During this stage, the lesion becomes semisolid and there is ring enhancement of the lesion but with less surrounding edema. The last stage is the nodular calcified, in which the lesion is calcified making it easier to see in CT and hyperintense in T2 weighted MRI sequences.[5,14,16]

Parenchymal disease is the most common location and easily seen on MRI. The second most common site with up to 20% of the lesions is the intraventricular form of the disease.[6,14,16] Ventricular lesions are known to have worse prognosis as they cause an inflammatory reaction producing ependymal inflammation, scarring, obstruction and ventriculitis. This phenomenon is known as granular ependimitis.[2,6,8,16] Fourth ventricular and occipital horns are the most common location for the intraventricular cyst.[2,3,7,8] Intraventricular cystic lesions are often missed by CT because they are isodense to CSF and the cysts do not enhance with contrast.[8,16] Occasionally, the cysts become evident on CT in patients with small ventricles where the lesion can cause mass effect on the parenchyma.[9] MRI is the preferred imaging modality due to the absence of bone artifact in the posterior fossa, multiplanar imaging and better resolution. MRI reveals 50–80% of the intraventricular lesions.[6,8,11,14,16] T2-FLAIR and T1 with contrast sequences are preferred for intraparenchymal lesions showing surrounding edema and a bright nodule within a cyst “hole-with-dot”. Some intraventricular lesions have ring enhancement features on T1 with contrast sequences and can cause edema at the attachment site.[10]

CT ventriculography has been previously described to diagnose intraventricular lesions.[2,4,15] Contrast injection into the CSF spaces is a safe technique that has been available for decades. Previous authors have described ventricular puncture or accessing shunt reservoir followed by CT. Our series describes the use of the CT ventriculography technique with injection of iohexol-240 via ventriculostomy catheter in patients where the usual imaging failed to demonstrate the number and characteristics of lesions. Iohexol-240 is a low osmolar, nonionic, iodinated contrast agent approved for intrathecal use and is absorbed from the CSF into the blood stream. It is eliminated via renal excretion. No metabolism, deiodination, or biotransformation occurs. This technique is rarely used in neurosurgery as non invasive MR is widely available. On the other hand, MRI can miss up to half of the intraventricular lesions.[6,8,11,14,16] The direct injection of iohexol-240 into the ventricle has the advantage of outlining these lesions and revealing them on an immediate CT. This technique can be used to detect lesions and plan interventions to treat the intraventricular form of the disease. Limitations include the following: being an invasive test, the need for trained personnel to perform the injection due to possibility of infection, increase in ICP, the timing of the injection and scanning, and the interpretation of the imaging.

## CONCLUSION

Intraventricular neurocysticercosis is a common parasitic disease which can be difficult to diagnose despite CT and MRI. Knowing the exact location of the disease is instrumental in treatment and surgical planning. The authors used the CT ventriculography technique with injection of contrast via the ventriculostomy catheter in two patients where the usual imaging failed to reveal the lesions. This technique is a safe and useful tool in the imaging armamentarium when cystic intraventricular lesions are suspected.
